# Can Family Motivation Enhance Men’s Uptake of Cascade Screening for Familial *BRCA1/2* Mutations?

**DOI:** 10.1177/15579883251343962

**Published:** 2025-08-07

**Authors:** Giulia Ongaro, Serena Petrocchi, Mariarosaria Calvello, Bernardo Bonanni, Irene Feroce, Gabriella Pravettoni

**Affiliations:** 1 Applied Research Division for Cognitive and Psychological Science, European Institute of Oncology (IEO), IRCCS, Milan, Italy; 2 Department of Psychiatry and Behavioral Science, Memorial Sloan Kettering Cancer Center (MSK), New York, NY, USA; 3 Division of Cancer Prevention and Genetics, European Institute of Oncology (IEO), IRCCS, Milan, Italy; 4 Department of Oncology and Hemato-Oncology, University of Milan, Italy

**Keywords:** *BRCA1*/2 mutation, genetic testing, cascade screening, cascade testing, men’s cancer prevention

## Abstract

Pathogenic variants in the *BRCA1* and *BRCA2* genes increase the relative and absolute risks of developing breast, ovarian, prostate, and pancreatic cancer. Clinical guidelines recommend cascade screening (CS) to enhance the identification of at-risk relatives. Despite the benefits of CS in facilitating access to appropriate cancer screening and risk-reduction strategies, CS uptake remains relatively low, particularly among at-risk men. Men’s decisions regarding CS appear to be driven more by familial rather than individual disease risk, framing the decision as a family duty. Little is known about the motivational factors that could encourage men’s participation in CS. This randomized controlled trial aimed to evaluate the effectiveness of two first-person, gain-framed messages in promoting CS intention among at-risk men: one featuring a self-referred narrative (SM) and the other a family-referred narrative (FM). A total of 110 male first-degree relatives of female *BRCA1/2* carriers were randomized into two groups. *T*-tests revealed no significant difference between groups in perceived message quality. Additionally, after controlling for age, the type of message received did not significantly influence participants’ levels of intention to undergo CS. These findings highlight the need for further exploration of the complex motivational factors influencing at-risk men’s adherence to CS. Future research should consider alternative health communication strategies tailored to different motivational drivers.

## Introduction

Germline pathogenic or likely pathogenic variants (PVs) in the *BRCA1* and *BRCA2* (*BRCA*) genes increase the relative and absolute risks of breast, ovarian, and other cancers, such as prostate and pancreatic cancer (Garutti et al., 2023). These variants follow an autosomal dominant inheritance pattern, meaning that first-degree relatives (FDRs) of a *BRCA* PV carrier have a 50% chance of being carriers themselves (Petrucelli et al., 2022). Clinical guidelines recommend cascade screening (CS), a process involving genetic counseling and testing for blood relatives of identified carriers, to improve the detection of at-risk individuals and enable genetically informed disease prevention (American College of Obstetricians and Gynecologists, 2018). Notably, younger and cancer-unaffected relatives derive the greatest health benefits from this approach (O’Neill et al., 2021). Despite the advantages of CS, which include facilitating appropriate regular cancer screening and risk-reduction strategies (such as, in females, prophylactic mastectomy or salpingo-oophorectomy for breast and ovarian cancer, respectively), adherence rates among at-risk family members remain low, with an overall uptake of less than 30% (Griffin et al., 2020). This gap is particularly pronounced among at-risk men, who participate at significantly lower rates than women, with an estimated male-to-female participation ratio of approximately 1:10 (Childers et al., 2018).

The literature suggests that several psychological and sociocultural barriers may interfere with men’s decision-making process regarding CS (Griffin et al., 2020). Men in *BRCA*-positive families are often minimally involved in communication, counseling, and testing procedures (Finlay et al., 2008; Roberts et al., 2018; Shiloh et al., 2013). This limited engagement is largely due to the historical association of *BRCA* PVs with women, reinforcing the misconception that *BRCA*-related cancer risks are exclusively a “female matter” (McAllister et al., 1998). Recent efforts have been made to raise awareness and promote the dissemination of genetic testing to males, aiming to rectify this gender bias and encourage greater male involvement in genetic counseling and testing processes (Dean et al., 2025; Ongaro et al., 2022; Peshkin et al., 2021; Petrocchi et al., 2022; Pritchard, 2019). Despite these initiatives, men still tend to employ distancing and avoidant coping strategies to manage the uncertainty arising from familial risk status (Lodder et al., 2001). This tendency may be further reinforced by the limited availability of risk-reducing interventions for men who test positive for a *BRCA* PV. While such coping strategies can help maintain emotional balance and reduce distress, they may also contribute to men’s reluctance to engage in discussions or take action regarding genetic testing and counseling.

According to the Uncertainty Management Theory (Brashers et al., 2001), individuals’ appraisal of their *BRCA*-related cancer risks significantly influences their decisions about genetic testing. Applying this framework, Rauscher et al. (2019) analyzed qualitative data from both tested and untested men in *BRCA*-positive families. Their findings indicate that men often perceive their uncertainty as irrelevant, making them less likely to seek information or actively engage in genetic testing. When familial uncertainty was perceived as a potential threat, men were more likely to seek information from relatives and pursue genetic testing for themselves and their family members. Men’s decisions regarding CS appear to be primarily driven by familial rather than individual disease risk (Hesse-Biber, 2018). This motivation is often framed as a family duty, emphasizing a sense of responsibility toward their relatives (Rauscher et al., 2019).

These findings predominantly rely on data from men who have already undergone testing and tested positive for *BRCA* PVs. The extent to which men who have not been tested might differ in their understanding of genetic cancer risks and their decision-making processes in the context of CS remains unclear. Little is known about the motivational factors that may support CS uptake among high-risk men.

Health communication researchers have extensively documented the effectiveness of narratives in promoting health behavioral intentions compared to non-narrative approaches (Braddock & Dillard, 2016; Zebregs et al., 2015). A crucial element of storytelling is the perspective from which a narrative is presented, as it significantly influences how the audience engages with the message. First-person narratives are conveyed from the protagonist’s perspective, providing direct insight into their emotions and inner thoughts during the narrative event. In contrast, third-person narratives are told by an external observer, who may or may not explore the protagonist’s internal experiences (van Peer & Maat, 2001). Research suggests that first-person narratives tend to be more effective than third-person narratives in reinforcing self-identity and facilitating the assimilation of key themes (van Peer & Maat, 2001). Moreover, health messages can be framed to emphasize either the benefits (gains) of adhering to a recommended behavior or the risks (costs) of non-adherence (Kim & Lee, 2017). While the effectiveness of message framing in cancer prevention and diagnosis remains debated (Ainiwaer et al., 2021), research on disease prevention—particularly regarding preventive behaviors—suggests that gain-framed messages may be more persuasive than loss-framed messages (Gallagher & Updegraff, 2012; O’Keefe & Jensen, 2007). Additionally, intentions are considered a key predictor of behavior (Schwarzer & Luszczynska, 2008) and are commonly used as a standard measure of health message effectiveness, alongside attitudes and actual behaviors (Ainiwaer et al., 2021).

Therefore, this study aims to assess the effectiveness of two first-person, gain-framed messages, in promoting the intention to undergo CS. These messages emphasize either the individual benefits or the family-related benefits of CS uptake. The hypothesis suggests that messages highlighting family benefits will be more effective in increasing the intention to undergo CS for *BRCA* PVs compared to those focusing on individual benefits, particularly among high-risk men.

## Methods

### Recruitment and Procedure

This study is a component of a broader longitudinal clinical trial investigating the psychological determinant of men’s CS uptake for familial *BRCA* PVs (ClinicalTrials.gov Registry: NCT04683068). The study received approval from the Ethics Committee of the European Institute of Oncology (IEO) under the approval number R1249/20-IEO 1314. As part of the study protocol (Ongaro et al., 2022; Petrocchi et al., 2022), the Division of Cancer Prevention and Genetics (IEO) maintains a registry. All female carriers with at least one documented germline pathogenic (C5) or likely PV (C4) in either *BRCA1* or *BRCA2* genes and having at least one male FDR have been contacted via phone/email and briefed about the research objectives and procedures. These women were requested to share this information with their male relative(s) and invite them to participate in the research. Upon the agreement of male relatives to participate and after completing informed consent, a questionnaire, implemented on the Qualtrics™ Platform, was sent via a link through email. Inclusion criteria include being male relatives of female patients affected by cancer (prevalently breast cancer) who underwent germline genetic testing and resulted in carriers of at least one PV in *BRCA* genes, being aged 18 or older, being able to provide informed consent, and being proficient in Italian. Exclusion criteria include prior personal cancer diagnoses (e.g., breast, pancreatic, or prostate cancer) and prior germline genetic testing for *BRCA1* and/or *BRCA2* PVs.

The study employed a randomized control trial design, where participants were randomly assigned to one of two conditions: receiving a self-referred narrative message (SM) or a family-referred narrative message (FM). Subsequently, participants responded to a manipulation check to confirm their comprehension of the message and reported their outcome expectations. Three weeks after completing the initial survey, a second link was sent via email to assess their intention to undergo genetic testing for the known familial *BRCA* PVs (T1). Participants’ data were pseudo-anonymized, and data collection was conducted using an ID code consisting of a combination of letters and numbers.

## Measures

### Pre-Test Evaluation

#### Demographic

Self-reported age, education level, occupation, parental status, and degree of kinship with the female carrier were recollected.

#### Health Status

Participants’ general health and existing diagnoses of chronic conditions were assessed using single-item measures (Shim et al., 2006). General health conditions were rated on a 5-point Likert scale ranging from “very poor” to “very good.” Existing diseases were coded as binary responses (“no” or “yes, specify”).

### Stimuli Messages

In the gain-framed condition, the main character adhered to the recommended behavior and experienced positive outcomes (Gray & Harrington, 2011). The message, delivered in the first person, emphasized the benefits of undergoing genetic testing to detect *BRCA* PVs. In the SM condition, the main character described why undergoing genetic testing was important for him, emphasizing potential individual benefits, such as adopting preventive health behaviors. In the FM condition, the structure of the message remained the same, but the main character focused on the benefits for his family, explaining how his decision helped reduce familial uncertainty and why it was important for his relatives. The narrative perspective was not manipulated across conditions. See Table 1 for message details.

**Table 1.** Message Texts

SM (221 words)	FM (226 words)
My name is Matthew and some time ago my sister was diagnosed with breast cancer. After a genetic test, she was found to be a carrier of a genetic mutation in a *BRCA* gene There are two *BRCA* genes: *BRCA1* and *BRCA2*. The presence of a mutation in one of these genes is associated with a higher risk of developing cancer in some organs (breast, ovary, pancreas, and prostate) than in those without the mutation	My name is Matthew and some time ago my sister was diagnosed with breast cancer. After a genetic test, she was found to be a carrier of a genetic mutation in a *BRCA* gene. There are two *BRCA* genes: *BRCA1* and *BRCA2*. The presence of a mutation in one of these genes is associated with a higher risk of developing cancer in some organs (breast, ovary, pancreas, and prostate) than in those without the mutation
Both men and women can have *BRCA1* or *BRCA2* mutations. These mutations can be passed on from parents to children	Both men and women can have *BRCA1* or *BRCA2* mutations. These mutations can be passed on from parents to children
Having a mutation does not necessarily mean developing a tumor, but having a greater predisposition to develop it	Having a mutation does not necessarily mean developing a tumor, but having a greater predisposition to develop it
I decided to undergo a genetic test because I think it’s important to me. They took my blood, got DNA from the sample, and detected the presence of the previously identified mutation in my family	I decided to undergo a genetic test because I think it’s important to my family. They took my blood, got DNA from the sample, and detected the presence of the previously identified mutation in my family
What are the advantages of genetic testing? Whilst I discovered that I have a higher risk of developing certain forms of cancer in the future, the advantage of genetic testing and knowing that I have a mutated gene is that now I can implement preventive behaviors (more controls, lifestyle change)	What are the advantages of genetic testing? Whilst I discovered that I have a higher risk of developing certain forms of cancer in the future, the advantage of genetic testing and knowing that I have a mutated gene is that now my family can implement preventive behaviors (more controls, lifestyle change)
I think my health has benefited from the decision I made to undergo the test and from the identification of the mutation	I think the health of my family has benefited from the decision I made to undergo the test and from the identification of the mutation

*Note*. SM = self-referred narrative message; FM = family-referred narrative message.

### Post-Test Evaluation

#### Manipulation Check

Two multiple-choice items were designed to assess whether participants had read and understood the message content. Each item included one correct answer and two incorrect distractors. Participants who failed to answer these questions correctly were excluded from the analyses.

#### Perceived Quality of the Message

Three items assessed the credibility, persuasiveness, and overall convincing nature of the message. Responses were collected using a 7-point Likert scale, ranging from “completely disagree” to “completely agree”. The scale demonstrated moderate internal consistency (α = .722, *r*s > .238).

#### Positive Outcome Expectation

Eight items were developed to measure self-referred positive outcome expectations (SOE) and family-referred positive outcome expectations (FOE) regarding genetic testing. Examples include statements like “*Genetic testing for the known familial BRCA mutation would increase my sense of safety about my health*” (SOE) and “*If I undergo the genetic test for the known familial BRCA mutation, my family members will have important information for their health*” (FOE). Participants rated their agreement on a 5-point Likert scale, ranging from unlikely to likely. Two total scores were computed, representing the average rating across all items in each subscale, with higher scores indicating stronger self- or family-referred outcome expectations. Reliability analysis yielded a Cronbach’s alpha coefficient of .857 for the overall scale (SOE: α = .752, *r*s > .291; FOE: α = .868, *r*s > .585).

#### Intention to Undergo Genetic Testing

The intention to undergo genetic testing was measured using three items evaluating participants’ inclination to engage in the behavior (Kim & Lee, 2017). An example of an item was: “*Do you intend to schedule genetic screening in the next few months?*” Responses were collected on a 5-point Likert scale, ranging from very unlikely to very likely. An overall intention score was calculated as the mean of the scores assigned to each item, with higher scores indicating a stronger intention. The scale demonstrated moderate internal consistency (α = .694, *r*s > .324).

### Data Analysis

Data analysis was performed using SPSS statistical software (Version 28.0- IBM). The data were normally distributed, and there were no missing values. Descriptive analyses were conducted to explore the socio-demographic characteristics of the sample. Independent samples *t*-tests and chi-square tests were used to examine systematic differences in the distribution of socio-demographic variables between the two groups. Analysis of covariance (ANCOVA) was employed to assess the effect of group assignment on intention level, while controlling for the socio-demographic characteristics of the sample.

## Results

### Participants

To our knowledge, no previous research has assessed the effectiveness of messages in promoting men’s intention to uptake CS, making it difficult to determine the effect size for sample size estimation. Therefore, we conducted a priori power analysis, using a conservative partial η^2^ of .07 and a power of 0.80, which indicated that a sample size of *N* = 107 would be required to detect a significant effect. The current sample (*N* = 110) should be adequately powered to detect effects of at least this magnitude (i.e., partial η^2^ = .07), which corresponds to a small-to-medium effect size. A total of 206 female *BRCA1/2* PV carriers were contacted for the study. Some carriers either declined participation (*N* = 46), had no eligible male FDRs (*N* = 45), or agreed to share information, but their male FDRs ultimately declined to participate (*N* = 21). Among the 131 identified and eligible male FDRs, a total of 110 (55 per group, SM and FM) were included in the analysis. All were male FDRs of female cancer-affected carriers. Participants’ ages ranged from 18 to 81 years (*M* = 41.33, *SD* = 17.21). Most participants were employed and well-educated. About half had children, with a number ranging from 1 to 6 (*M* = 1.9, *SD* = 0.99). Regarding self-reported health status, most participants rated their health as good or very good (63.6%), while 30% reported chronic conditions such as diabetes or hypertension. Additional descriptive statistics are provided in Table 2.

**Table 2.** Characteristics of the Sample

Variables	Self-referred narrative message group (*N* = 55)		Family-referred narrative message group (*N* = 55)		*p* Value
	Frequency	%	Frequency	%	
Employment status					
Employed	41	74.5	44	80	
Not employed	14	25.5	11	20	.449
Education level					
Primary/secondary school	14	25.5	6	10.9	
High school	20	36.4	33	60	.682
University	21	38.2	16	29.1	
Parental status					
With children	30	45.5	21	38.2	.087
Without children	25	54.5	34	61.8	
Degree of kinship with the proband					
Brothers	24	43.6	23	41.8	
Sons	19	34.5	28	50.9	
Fathers	8	14.5	0	0	.072
More than one family member	4	7.3	4	7.3	

### Messages Exposure and CS Intention

*T*-tests revealed no significant differences between the two groups in the perceived quality of the message (*t*_(108)_ = −0.807, *p* > .05). Specifically, both groups found the message to be moderately credible, convincing, and persuasive (SM: *M* = 5.26, *SD* = 0.92; FM: *M* = 5.40, *SD* = 0.88). Furthermore, *t*-tests indicated no significant differences between the SM and FM groups in SOE (*t*_(108)_ = −0.728, *p* > .05) or FOE (*t*_(108)_ = 0.628, *p* > .05).

Despite randomization, *t*-tests identified a significant difference in age between the SM and FM groups (*t*_(108)_ = 2.811, *p* = .006), with the SM group being older (*M* = 45.84, *SD* = 18.56) than the FM group (*M* = 36.89, *SD* = 14.58). ANCOVA results showed that age was significantly associated with participants’ intention to uptake CS (*F*_(1, 96)_ = 4.82, *p* = .031). However, after controlling for age, there was no significant effect of message type on intention levels (*F*_(1, 96)_ = 1.01, *p* > .05). Specifically, participants’ intention levels did not differ based on the message they received (SM: *M* = 3.21, *SD* = 0.93; FM: *M* = 3.41, *SD* = 0.81).

## Discussion

This research aimed to evaluate the effectiveness of two gain-framed messages, presented in the first person, in encouraging CS uptake. These messages emphasized the benefits of CS uptake for either the individual or their family. We hypothesized that messages highlighting family-related benefits would be more effective in increasing CS uptake intentions among *BRCA* PV carriers, particularly among men at high-risk of *BRCA* PVs, compared to messages focusing solely on individual benefits.

Several qualitative studies examining the experiences of men in *BRCA*-positive families suggest that men and women may have different motivations for undergoing genetic screening (Daly et al., 2003; Lodder et al., 2001; Peshkin et al., 2021; Shiloh et al., 2013). These studies indicate that men often perceive genetic testing as a familial responsibility, choosing to undergo screening primarily for the benefit of their family members, especially their offspring. However, contrary to our predictions, our findings did not reveal significant differences in the effectiveness of messages emphasizing individual versus family benefits in promoting men’s intention to uptake CS. In other words, familial and individual incentives appeared to have a similar impact on men’s intentions regarding *BRCA* PV screening. The two personalized messages did not produce variations in inclination toward genetic screening within this underexplored population. Based on previous qualitative research (Dean et al., 2020; Hallowell et al., 2005; Rauscher et al., 2018) we initially expected that a family-centered approach would be more effective in encouraging men to undergo genetic screening than an individual-focused approach. However, prior studies primarily explored the motivations of men who had already undergone *BRCA* genetic testing, often within genetic counseling settings (Hallowell et al., 2005; Rauscher et al., 2019). Genetic counselors play a crucial role in supporting informed decision-making by providing key information, resources, and testing options, while also helping individuals navigate the complexities of a family history of cancer, aiming to enhance patients’ understanding of test results and their familial implications. Previous research may not fully capture the intrinsic motivations for testing, as these decisions could have been influenced by counseling consultations. Our findings suggest that untested men without prior genetic counseling may have limited awareness of the familial implications of genetic information, which could explain the limited effectiveness of the tailored family-focused messages. Genetic counseling serves multiple functions beyond education, including enhancing understanding and mitigating emotional distress. These aspects may significantly influence individuals’ decision-making processes and responses to tailored messages.

Limitations of the study should also be acknowledged, as they may help explain the lack of differential effects between the two tailored messages. The exclusive use of a gain-framed approach may have contributed to their ineffectiveness in eliciting distinct levels of adherence intention. Research suggests that when individuals encounter first-person narratives, the framing effect may depend on their current stage of change (Kim & Lee, 2017). While we did not have precise information on participants’ stage of change, it is reasonable to infer that, given their lack of prior genetic counseling or contact with the Division of Cancer Prevention and Genetics (IEO) or other genetic testing facilities, they were likely in a pre-contemplative or contemplative stage—both pre-intentional phases, where individuals are either unaware of the need for change (pre-contemplation) or beginning to consider it without yet committing to action (contemplation) (Prochaska & Di Clemente, 1982; Prochaska et al., 1992). In smoking cessation research, a loss-framed first-person narrative has been shown to increase quit intentions and stage progression among pre-contemplative smokers compared to a gain-framed message (Kim & Lee, 2017). However, logistical challenges, including the difficulty in recruiting participants, prevented us from testing an alternative loss-framed message. Future studies should address this limitation by expanding the sample size and investigating the differential effectiveness of gain- and loss-framed messages based on participants’ stage of change in high-risk men. Moreover, the sample size, although statistically powered, may still have been insufficient to detect subtle differences in intention between groups. Additionally, the effect of the messages on CS intention was assessed longitudinally, specifically at 3 weeks post-intervention. Message retention is critical for sustaining the intervention’s impact (Suka et al., 2020), but this study did not measure retention at 3 weeks. Research suggests that message repetition can enhance retention and strengthen responses to tailored messages (Cacioppo & Petty, 1979; Shi & Smith, 2016). To improve the sustainability of messaging strategies encouraging *BRCA* genetic testing, future studies should examine whether repeated exposure to messages about *BRCA* PVs implications improves intention to uptake CS.

Finally, it is important to acknowledge the relatively limited focus on men in *BRCA* awareness campaigns. This lack of targeted outreach may have influenced the effectiveness of the messages. Future research should explore men’s awareness levels and factors associated with greater awareness, such as a significant family history of cancer. Increasing awareness is essential to inform men about their role in safeguarding not only their health but also the well-being of their families, ultimately enhancing their understanding of associated risks.^25^
